# An Interdisciplinary Management of Avulsed Maxillary Incisors: A Case Report

**DOI:** 10.7759/cureus.23891

**Published:** 2022-04-06

**Authors:** Revathy Parthasarathy, Srividhya Srinivasan, Vikram C, Yashini Thanikachalam, Anupama Ramachandran

**Affiliations:** 1 Conservative Dentistry and Endodontics, Chettinad Dental College and Research Institute, Chennai, IND; 2 Periodontics, Chettinad Dental College and Research Institute, Chennai, IND

**Keywords:** traumatic dental injury, replantation, gbr, cbct, avulsion

## Abstract

The current case report presents a case of a road traffic accident comprising dental avulsion of maxillary incisors. A 30-year-old male reported missing teeth following trauma in his upper front tooth region. The avulsion of maxillary right central and lateral incisors along with laceration in upper and lower lips was evident. The avulsed teeth were stored in milk after a brief extra-oral dry time of one hour and were carried to the department with a one-hour delay. After clinical and radiographic investigations, no mobility or fractures were apparently noticed. The avulsed 11 and 12 had closed apices with intact crown and root surfaces. Following an extra-oral endodontic therapy, immediate replantation was scheduled. Subsequently, the alveolar sockets were irrigated with saline to dislodge the clot and any gross debris that was present. Teeth were reimplanted into the socket after placement of PRF membrane around the root surface. A semi-rigid wire and composite splinting were done for a span of two weeks and antibiotics were prescribed. An exploratory CBCT taken revealed a cortical bone loss in the buccal region of 12 and 11. An interdisciplinary approach to regenerate the osseous defect was then carried out. A follow-up of two weeks, four weeks, three months, six months, and a year was done. Evaluation performed after a year revealed the absence of clinical symptoms and satisfactory healing with no signs of resorption radiographically.

## Introduction

Traumatic injuries of the dentition are frequently seen in children and young adults in which luxative injuries are seen in primary dentition and crown fractures are commonly seen in permanent dentition. Among the traumatic dental injuries (TDI), avulsion comprises 0.5 to 16% of permanent teeth [1,2].

According to Andreasen et al., avulsion is defined as the complete displacement of a tooth from its socket in alveolar bone owing to trauma. In permanent teeth, avulsion occurs commonly in the maxilla due to fights and sports injuries. Maxillary central incisors are widely affected and commonly seen in the age group between seven and nine years [3].

Incomplete root maturation and reduced resistance to extrusive forces by the alveolar bone/periodontal ligament (PDL) at the time of eruption are the two major etiological factors of avulsion [4,5]. The treatment option for an avulsed tooth depends upon the development of the root apex and the condition of the PDL cells. The PDL cell condition in turn depends upon the extra-oral dry time and storage medium used.

Replantation is considered to be the treatment of choice for an avulsed tooth since it maintains the aesthetic and function of the patient [2]. Although few replanted teeth have shown less long-term survival rate, a recent study has proved the long-term success of replanted teeth following the International Association of Dental Traumatology (IADT) treatment guidelines [6].

An ideal storage media for an avulsed tooth must maintain the function and viability of the PDL cells, so that the fibroblasts of the PDL cells repair the damaged root surface. Storage media for an avulsed teeth are broadly classified as artificial and natural media. Natural storage media like coconut water, milk and saliva are more readily available than artificial medias like Viaspan, Hank’s Balance Salt Solution. The pH (6.5-6.8) and osmolality (250mOsm) of the storage media should be compatible with the PDL cells and it must also contain nutrients required for cell growth [7].

Extra-oral dry time plays a vital role in determining the outcome of avulsed teeth. An extra-oral dry time of more than 60 minutes has deleterious effects on the PDL cells like decrease in the regenerative ability and resorption of the root [2]. Thus, the current case report emphasizes the importance of inter-disciplinary approach in reimplanting the avulsed teeth despite the increased extra-oral time of two hours.

## Case presentation

**Figure 1:**
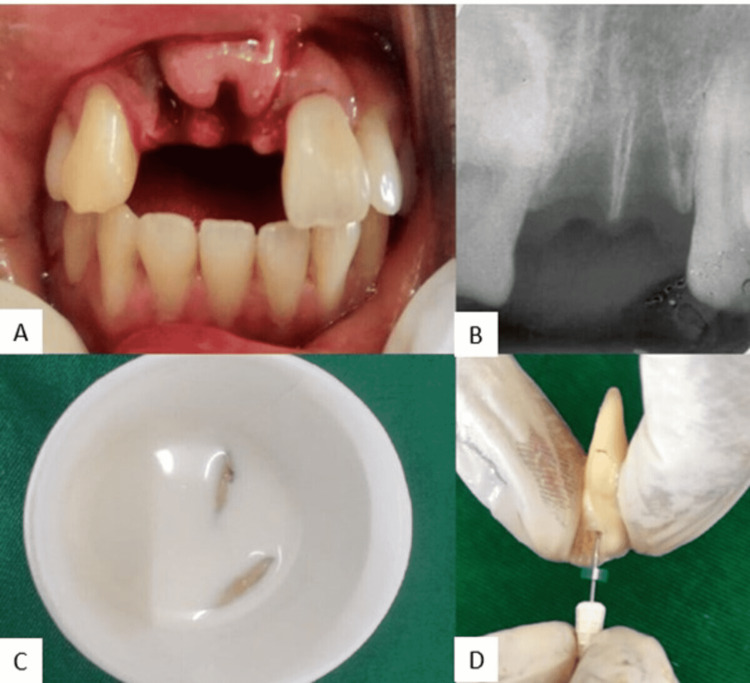
(A, B) Pre-operative clinical and radiographical images with avulsed socket 11 and 12. (C) Avulsed teeth stored in milk. (D) Extra-oral root canal treatment initiated.

A 29-year-old male patient reported to the Department of Conservative Dentistry and Endodontics with trauma due to a road traffic accident in his maxillary anterior region. The patient was however found to have no systemic ailments associated while obtaining the medical history. He was further evaluated for any other physical injuries and neurological disturbance following the trauma. The patient was conscious and well-oriented to place, time, and person during the time of examination. He had sustained an injury to his upper anterior teeth along with an associated soft tissue laceration present in both upper and lower lips. During the extra-oral examination, it was evident that the upper and lower lips were swollen as well as lacerated. During Intra-oral examination, the patient presented with the missing maxillary right central (11) and lateral incisors (12) (Figures 1A, 1B). Also, the anterior maxillary segment was inspected and palpated for detection of fracture and abnormalities if present. The patient had preserved the 11 and 12 in milk (Figure 1C) after a brief extra-oral dry time of an hour which was carried to the department after one and a half hours. In order to rule out any other concomitant luxation injuries to adjacent teeth and their associated alveolar structures, an Intra-oral Periapical radiograph was advised in the maxillary anterior region. An orthopantomography (OPG) or cone beam computed tomography (CBCT) was not suggested, as the immediate replantation of avulsed teeth was considered most crucial. The patient was explained the potential complications of replanting the avulsed tooth which sustained a dry extra oral period of one hour. Once the Informed consent from the patient was obtained, it was planned to reposition the 11 and 12. The avulsed 11 and 12 were checked for debris accumulation on the root surface and fractures. Both 11 and 12 had well intact crown portions and the root was fully formed. After taking the teeth from the storage media (milk), it was washed gently with saline and then the PDL remnants adhering to the root surface were gently scraped with the help of a Bard-Parker No.11 blade. An IntraOralPeriApical (IOPA) of 11 and 12 were taken prior to the initiation of extra-oral endodontic therapy in order to determine the root canal morphology (Figure 1D).

**Figure 2:**
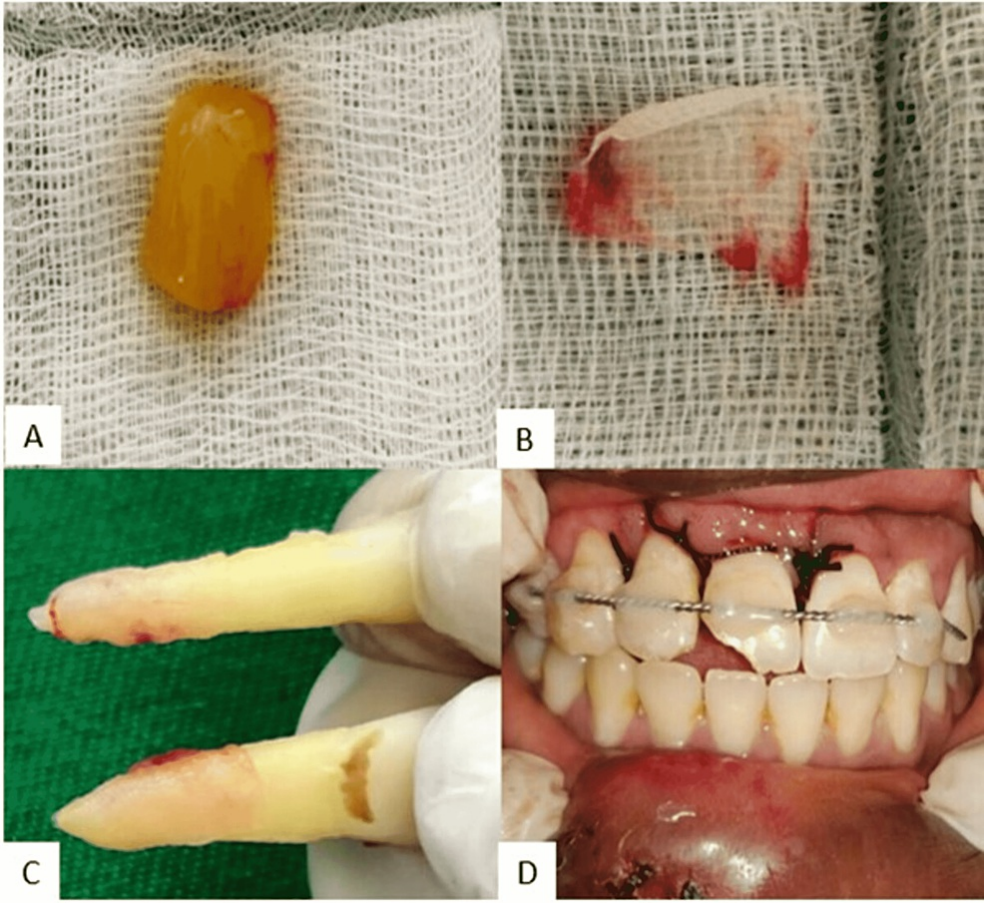
(A) Platelet-rich fibrin (PRF). (B) PRF membrane. (C) PRF membrane wrapped around the roots of 11 and 12. (D) Avulsed teeth re-positioned and splinting done with orthodontic wire and composite.

Access opening and pulp extirpation was done. The canal was well cleaned with ethylenediamine tetra-acetic acid (EDTA), saline and povidone iodine. Rotary Protaper gold file system (Dentsply Tulsa Dental; Tulsa, Oklahoma) was used for the mechanical shaping of canal. An intracanal medicament of calcium hydroxide was placed inside the root canal and access opening was sealed with Glass Ionomer cement. Root canal obturation alone was deferred, to reduce the extra-oral duration of the avulsed incisors. 1.23% Acidulated phosphate fluoride (APF) was used for 15 minutes to treat the root surface. Meanwhile, 10 ml of patient’s venous blood was collected, and centrifugation procedure was carried out to obtain platelet-rich fibrin (PRF) (Figure 2A). The membrane obtained after pressing the PRF with the sterile gauze was wrapped around the root surface of the avulsed teeth (Figures 2B, 2C). Local anesthetic infiltration of 2% lignocaine was given in maxillary anterior region. The socket of 11 and 12 was sufficiently debrided with povidone iodine without distortion of the coagulum. The sockets received the avulsed teeth back with gentle digital pressure and was secured with the help of wire and composite splint. The patient was advised to remain with the splint for a period of two weeks (Figure 2D).

**Figure 3:**
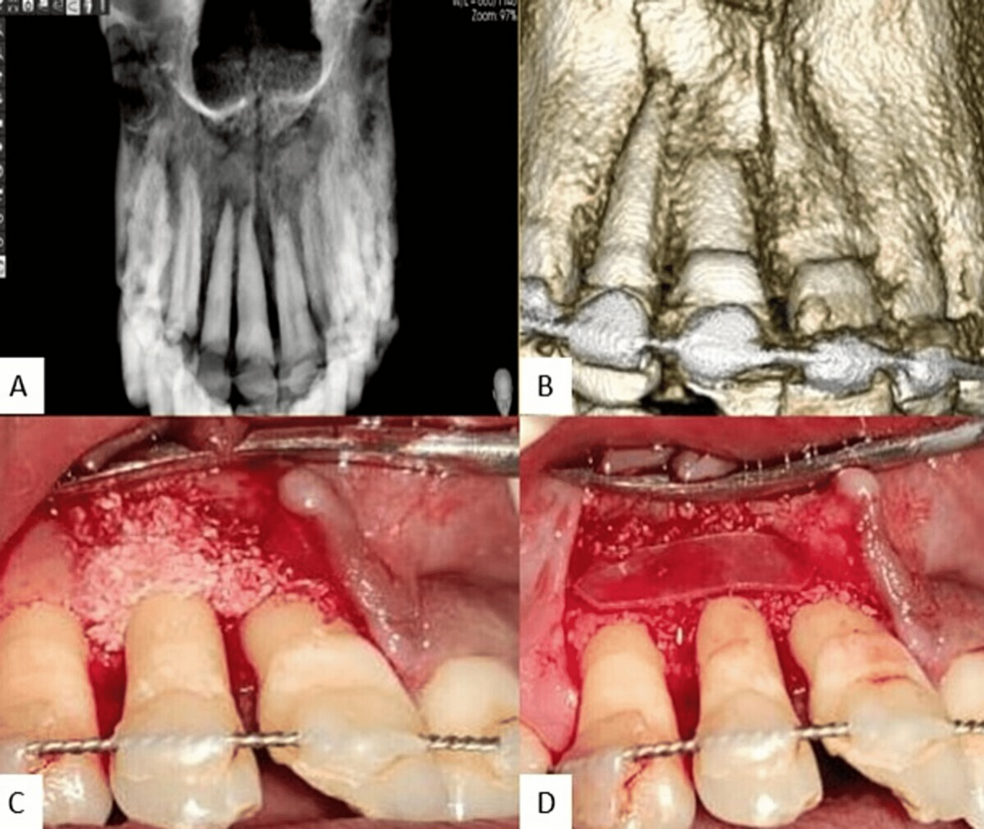
(A, B) Exploratory CBCT reveals labial cortical bone loss in relation to 11 and 12. (C) Inter-disciplinary management of cortical bone loss by placement of bone graft. (D) Placement of GTR membrane. CBCT - cone beam computed tomography, GTR - guided-tissue regeneration

Oral antibiotics were prescribed for the patient for five days and the importance of anti-tetanus booster was emphasized. After two weeks, the obturation of 11 and 12 was done with gutta percha and the access cavity was sealed with light cure composite. On exploratory CBCT taken after two weeks, a loss of labial cortex was evident in 11 and 12 region (Figures 3A, 3B) and so the patient was referred to the Department of Periodontics for an opinion and management of bone loss in the maxillary anterior region.

Upon periodontal examination, clinical attachment loss of 10 mm was evident and regenerative periodontal surgery was advised. A vertical incision was given, and a full thickness muco-periosteal flap was elevated in relation to 11-13. Bone graft (OSSEOGRAFT™ - Advanced Biotech, India) (Figure 3C) and guided tissue regeneration (GTR) membrane (HEALIGUIDE® - Advanced Biotech) (Figure 3D) were placed in the 11 and 12 regions. The flap was approximated using simple interrupted sutures.

**Figure 4:**
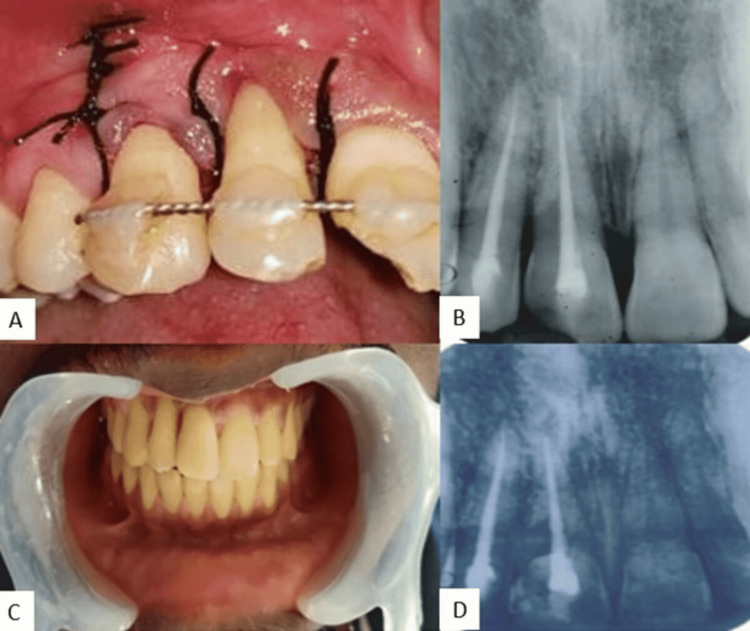
(A, B) Post-operative clinical and radiographic images of reimplanted 11 and 12. (C) Restorative management of fracture 11. (D) One-year follow-up radiographic image.

Consecutive to the periodontal regenerative procedures, splinting duration was extended further, for about five weeks (Figure 4A). Following five weeks, splinting was removed after obtaining positive periodontal outcome and Composite restoration was done in 11 to restore the fracture extending up to the dentin (Figures 4B, 4C). Patient was called for follow-up and radiographs were taken during third week, one month, three months, six months, and one year postop (Figure 4D). The PDL ligament space and the periapical structures appeared healthy and intact throughout the review visits.

## Discussion

Immediate replantation is considered an excellent treatment of choice for an avulsed permanent tooth owing to its ability to maintain the alveolar form, function, and PDL cell viability. In the current case, the immediate replantation of 11 and 12 was done following an extra-oral time of 2 hours. The literature states that replantation of avulsed teeth usually occurs one and four hours after trauma. 

Outcome and the rate of survival for avulsed teeth depend upon factors like extraoral dry time, storage media, and root defects if any are present [8]. An ideal storage medium maintains the PDL cell viability and prevents drying of the cells. Milk being an easily available natural storage medium contains a mixture of nutrients required for the PDL cells. Low fat and cold milk serve as better storage media. A systematic review suggests that natural storage media are more effective than artificial media, among which milk serves as the best because it is easily available and economical [9].

Treatment of the root surface of an avulsed tooth with fluoride is recommended to decrease the rate of root resorption. Treatment with 2% APF provides resistance to inflammatory and replacement root resorption because fluoride acts directly on dentin, cementum, and enamel converting the hydroxyapatite into fluoroapatite [10]. It also stops the clastic effect of the cells [11].

A delay in the replantation of avulsed teeth will lead to resorption of the root due to the presence of necrotic PDL, thus it is required to remove the dead PDL. Mechanical debridement is considered a simple and effective way of removing the PDL as it does not aggressively remove the tissue [12]. According to the IADT guidelines, after the mechanical debridement of the root, the avulsed teeth must be immersed in 2.4% APF for five minutes [13]. Hence in the current case, the roots of the avulsed 11 and 12 were mechanically debrided of PDL cells and were immersed in 1.23% APF before initiation of extraoral RCT.

Irrespective of the root treatment, the replanted teeth should be root canal treated. This is to remove the necrosed pulp tissues present inside the canal. In the present study, root canal treatment was initiated extra-orally, followed by placement of intracanal medicament, calcium hydroxide. Calcium hydroxide helps in reducing inflammatory resorption, counteracting the endotoxins [14], and alters the dentin making it less susceptible to dentinoclastic action [15]. Though the recent guidelines suggest long-term placement of calcium hydroxide for about one month, the same has equal effectiveness to that of short-term application, if the teeth are free of infection [16].

Following the placement of intracanal medicament, a temporary access filling was done. The root surfaces of the teeth were covered by a PRF membrane. PRF is obtained by centrifuging the patient’s own blood, and it does not require any anti-coagulants. The main advantage of platelet concentrates is their ability to improve wound healing by the presence of various growth factors. This growth factor improves the viability of PDL cells and also improves their differentiation and growth.

Stabilization of the avulsed teeth is of utmost importance as it prevents further damage to the pulp and periodontal tissues and aids in its healing [17]. Trope et al. suggested the use of semi-rigid splint using steel wire and composite for seven to 10 days whereas, recent studies states that short term application of passive, flexible splints with stainless steel wire of 0.016” or 0.4 mm or with nylon fishing line allow physiologic movement of teeth thereby aiding in healing and avoiding ankylosis [18]. The ideal splinting period for a reimplanted avulsed teeth is said to be two weeks depending on the root maturation and length [2]. In case of avulsion along with loss of cortical bone, the splinting time period can be extended to about four to eight weeks [19]. In the present study, flexible splint with orthodontic wire and composite was done which was stabilized for two weeks, followed by extended splinting time of about five weeks after the management of cortical bone loss.

The usage of CBCT in treating TDI was first employed in the year 2007. The recent IADT guidelines suggests the use of CBCT in diagnosing the severity and type of traumatic injuries. In the current case report, following the emergency management, an exploratory CBCT was taken, to determine the severity of the injury, which revealed labial cortical bone fracture. To manage the bone interdisciplinary, approach was carried out by performing periodontal regenerative procedures.

GTR refers to the use of a barrier membrane at the interface with gingival/epithelial connective tissue, PDLs, and alveolar bone to facilitate periodontal tissue regeneration, while GBR pertains to the regeneration of alveolar bone. In the current case, the periodontal regeneration was achieved by placing bone graft followed by GTR membrane. This novel approach aids in angiogenesis, osteogenic migration and wound stabilization which promotes the formation of granulation tissue that is rich in vascularity and offers better healing [20].

Reimplanted avulsed teeth must be under continuous follow-ups at time intervals of two weeks, four weeks, six months, one year, and then every year later for at least five years [2]. Both clinical and radiographical examination should be done to evaluate the prognosis. In the current case report, a one-year follow-up was done and documented. Patient must be informed about the favorable or unfavorable outcomes of the treatment, and other treatment options must also be discussed.

## Conclusions

The patient’s knowledge about avulsion and its management is of utmost importance in the outcome of the avulsed teeth. Instructions on the transportation and storage of the avulsed teeth must be an important patient education as it plays a pivotal role in the treatment. Regardless of the time, replantation itself serves as the best treatment of choice as it maintains the form and function of the bone for later prosthetic needs if there might be any failure in the future.
